# Neonatal Natural Killer Cell Function: Relevance to Antiviral Immune Defense

**DOI:** 10.1155/2013/427696

**Published:** 2013-08-26

**Authors:** Yen-Chang Lee, Syh-Jae Lin

**Affiliations:** ^1^Department of Obstetrics/Gynecology, Chang Gung Memorial Hospital, Taoyuan 333, Taiwan; ^2^Division of Asthma, Allergy, and Rheumatology, Department of Pediatrics, Chang Gung Children's Hospital, College of Medicine, Chang Gung University, Taoyuan 333, Taiwan

## Abstract

Neonates are particularly susceptible to various pathogens compared to adults, which is attributed in part to their immature innate and adaptive immunity. Natural killer cells provide first-line innate immune reactions against virus-infected cells without prior sensitization. This review updates phenotypic and functional deficiencies of neonatal cells compared to their adult counterparts and their clinical implications.

## 1. Introduction

Natural killer (NK) cells are a distinct lineage of lymphoid cells defined by the expression of CD56 and NKp46 and by the absence of CD3, providing the first-line defense by lysing tumor and virus-infected cells in a nonmajor-histocompatibility-complex- (MHC-) restricted fashion without the need of prior sensitization [1–4]. Receptors commonly expressed on NK cell surface include killer immunoglobulin-like receptors (KIR), heterodimeric C-type lectin receptors which can be inhibitory (NKG2A) or activating (NKG2C and NKG2D), and natural cytotoxicity receptors (NCR) [5–8].

NK cells can perform antibody-dependent cellular cytotoxicity (ADCC) through CD16 [9] or directly exert their cytotoxic ability by the release of perforin and granzyme B [1, 3, 10]. NK cells also kill tumor and virus-infected cells by apoptosis, mediating through TNF-related apoptosis-inducing ligand (TRAIL) and FasL [11].

NK cells also produce many cytokines such as interleukin (IL)-5, IL-10, IL-13, GM-CSF, TNF-*α*, TGF-*β*, and IFN-*γ* [8, 12]. IFN-*γ* can induce T_H_1 responses and also up-regulate MHC-I expression on antigen presenting cells. Recent pieces of evidence suggest the greater regulatory roles for NK cells by bridging innate with adaptive immunity via their intimate interactions with dendritic cells, B cells, and T cells [13–15]. Human NK cells can be divided into two major subsets based on CD56 expression: the CD56^dim^ subset accounts for the majority (>90%) of peripheral blood NK cells that are more effective at mediating cytotoxic function, while the CD56^bright^ CD16^dim^ subset, characterized by the ability to produce immunoregulatory cytokines, constitutes only a minority (<10%) of the total NK cells [1, 8].

## 2. Immunophenotype of Neonatal Natural Killer Cells

Human neonates have comparable or higher numbers and percentages of NK (CD56^+^/CD16^+^/CD3^−^) cells in their peripheral blood compared to adults [16–18]. Gaddy and Broxmeyer showed that the CD56^−^CD16^+^ subset NK cells are more abundant in the neonates and are precursors of the more mature CD56^+^CD16^+^ NK cells [19]. The CD56^bright^ and CD56^dim^ NK cell subsets are present in similar proportions in neonatal blood and adult blood [20, 21]. Very few neonatal NK cells express CD57, a marker of terminal differentiation [21]. CD57^+^ NK cells are characterized by a higher cytotoxic capacity but decreased cytokine responsiveness [22]. Neonatal NK cells express lower L-selectin (CD62L) compared to adults [20, 23], highlighting their unique lymph node homing properties. We and others have found a lower level of CD54 expression on neonatal NK cells [24, 25], suggesting an impaired ability to adhere to target cells. We observed a higher NKp46 expression in neonatal NK cells compared to adults [26]. The level of expression of other triggering receptors like NKp30, NKG2D, and NKG2A/CD94 decreases with age [27, 28].

## 3. Neonatal Natural Killer Cytotoxic Function

We and others have shown that neonatal NK cells show less NK cell cytotoxicity and ADCC than their adult peripheral blood (APB) counterparts, respectively [9, 20, 29–31]. Several possibilities contribute to the impaired cytotoxicity of neonatal NK cells. First, neonatal NK cells form fewer NK-target cell conjugations compared with adult NK cells [21]. Secondly, compared with adult NK cells, neonatal NK cells express lower levels of adhesion molecules like L-selectin and CD54 [25, 32]. In contrast, the expression of inhibitory receptors, such as CD94/NKG2A, was higher on neonatal NK cells than those on adult NK cells [28]. Finally, neonatal NK cells exhibit an impaired F-actin polymerization in forming immunologic synapses with leukemic cells, a defect that could be reversed with IL-2 [33]. Interestingly, the level of expression of NK cytotoxic machinery such as perforin and granzyme B by neonatal NK cells was comparable to or even higher than APB NK cells [20, 33]. We observed that neonatal NK cells were less susceptible to K562-induced apoptosis than adult NK cells [34].

## 4. Cytokine Production of Neonatal NK Cells

NK cells serve as a bridge between innate immunity and adaptive immunity and release a variety of cytokines such as GM-CSF, TNF-*α*, and IFN-*γ* and chemokines like MIP-1*α*, MIP-1*β*, and RANTES that modulate the subsequent adaptive immune response. Krampera et al. reported that neonatal NK cells showed a lower percentage of TNF-*α* producing cells compared to adult NK cells [35]. We and others have shown that resting neonatal NK cells did not produce IFN-*γ* [26, 36]. However, neonatal NK cells exhibited higher IFN-*γ* production and CD69 expression than APB NK cells after stimulation with IL-12 and IL-18 [37].

## 5. Neonatal NK Cell Response to Viral Infections

NK cells play a critical role of controlling most viral infections [38]. NK cells emerge as an effective, early defense against viral infections by mediating cytotoxicity and cytokine production [39, 40]. As previously stated, the CD56^dim^ NK subset is more cytotoxic, while the CD56^bright^ subset mainly produces cytokines that serve as immuneregulators [8, 41].

A series of interactions between viruses and surface receptors on NK cells are critical in activating antiviral NK defense. The NCRs like NKp46, NKp30 and NKp44, and NKG2D are essential in NK cell activation, while the inhibitory NK receptors like p58.1, p58.2, p70, NKG2A, and LIR-1 provide negative signals that allow NK cells to discriminate between normal cells and cells that have lost their MHC-I molecules [42–44]. NKp46 is the major triggering receptor involved in the natural cytotoxicity of human NK cells and binds hemagglutinin of influenza virus [45, 46], while NKG2A and NKp30 are involved in interaction with dendritic cells that shape subsequent T-cell response [47, 48].

NK cells are activated during the initial stages of viral infections by cytokines and chemokines, including IFN-*α*, IFN-*β*, IL-12, IL-15, and IL-18, produced by infected cells or by activated dendritic cells and macrophages [49, 50]. Activated NK cells produce TNF-*α*, IFN-*γ*, GM-CSF, and chemokines that are significant in the noncytolytic control of viral infections and may help shape the subsequent adaptive immune response. The number of NK cells in the liver during mouse cytomegalovirus (CMV) infection was decreased in MIP-1*α*-deficient mice, suggesting that chemokines may play a crucial role in NK trafficking during viral infection [51]. The chemokine-dependent recruitment of NK cells during viral infection is mediated by IFN-*γ* [52].

The phenotypic and functional defects of neonatal NK cells may compromise their antiviral immune defense. Patients with primary NK cell deficiency are particularly susceptible to severe varicella and complicated herpes virus infections [53, 54]. Adult NK cells expressing an inhibitory receptor for self-MHC-I show enhanced effector function, the so called NK licensing [55–57]. NK cell repertoires in neonates are not yet shaped toward increased clonal frequency of KIR for self-MHC-I [58], contributing to their greater infection susceptibility.

Virus infection can induce modifications of the repertoire of NK cell receptors, notably CMV [59]. *In vitro* exposure to CMV leads to increased expression of the inhibitory receptors KIR2DL1 and KIR2DL3 only in CMV-seropositive donors [60]. Pediatric CMV infection is associated with the expansion of the CD94/NKG2C^+^ NK cell subset [61].

Increased NK cell activity has been correlated with protection from human immunodeficiency virus (HIV) infection in highly exposed seronegative subjects [62]. We and others have shown the deficient NK and ADCC function of NK cells in the HIV-infected subjects [63–65]. Different KIR-HLA associations may determine susceptibility to HIV infection and the rate of disease progression [66]. Ballan et al. observed an increased frequency of NK cells expressing inhibitory KIR in HIV-infected children. The increased expression of KIR2DL3, NKG2C, and NKp46 on NK cells correlated with decreased CD4^+^ T-lymphocyte count [67]. HLA-C/KIR interactions may play a role in maintaining the immunity in HIV-infected long-term nonprogressors [68]. Increased KIR2DL3 expression of NK cells was associated with a full immunological recovery after effective antiretroviral therapy in HIV-infected subjects [69].

Immune activation of HIV leads to enhanced CCR5 expression on NK cells [70]. The chemokines MIP-1*α*, MIP-1*β*, and RANTES produced by NK cells bind to CCR5 and inhibit HIV cell entry and replication [71]. Interestingly, Bernstein et al. reported that neonatal and adult NK cells produce comparable amounts of chemokines [72], while Jacobson et al. showed reduced expression of MIP-1*α*, MIP-1*β*, and RANTES in neonatal NK cells upon challenge of autologous HIV-infected CD4^+^ T cells [73].

Severe acute respiratory infections with influenza virus in infants are associated with reduced level of peripheral blood NK cells [74, 75]. We and others have shown that influenza virus directly infects NK cells and induces their apoptosis [26, 76]. We further showed that influenza-A-induced apoptosis in neonatal NK cells was more pronounced than in adult NK cells [26], which may contribute to the greater morbidity of influenza-infected infants less than 6 months of age [77, 78], despite the possible protection provided by maternal antibodies.

## 6. Interleukin-15 Enhances Neonatal NK Function

IL-15, mainly produced by macrophages and monocytes, uses the common *β* and *γ* chains with the IL-2 receptor and a unique *α* chain for binding and signal transduction [79]. IL-15 plays a pivotal role in NK differentiation and survival [80, 81]. The interaction of IL-15 with its receptor complex on NK cells leads to a series of signaling including activation of Janus kinase (Jak)/signal transducer and activator of transcription (STAT) pathways, similar to that of IL-2 [82, 83]. IL-15 expression is significantly decreased at the gene and protein levels in neonatal mononuclear cells compared to APB mononuclear cells [84]. We and others have shown that neonatal NK cells rapidly acquire cytotoxic activity after IL-15 stimulation, although this activity was still lower than correspondingly IL-15-treated adult NK cells [29, 31].

IL-15 is also capable of inducing antigen-independent expansion and differentiation of human naive and memory CD8^+^ T cells and enhances their replicative potential [85, 86]. Administration of IL-15 results in increased antimicrobial activity in mice against herpes simplex virus (HSV) infection [87, 88] and enhances HIV-specific CD8^+^ T-cell responses in HIV-infected subjects [89]. IL-15 may be used as an adjunct to antiretroviral therapy to bolster immune reconstitution in HIV-infected subjects [90]. The ability of IL-15 to activate both NK and CD8^+^ T cells makes it a potential effective immunotherapy for neonates with severe life-threatening viral infections.

## 7. Conclusion

Phenotypic and functional differences between neonatal and adult NK cells were highlighted in this paper. It is now known that different KIR-HLA combinations may modulate NK function and influence the progression of infectious diseases. NK cell repertoires of the neonates are not yet shaped toward increased clonal frequencies of KIR for self-HLA class I like that of the adults. The frequency of NK cells expressing cognate KIR for self-HLA class I may gradually increase from neonatal period to adulthood through certain viral infections. Further work will be needed to explore what triggers the transition from an unbiased neonatal KIR repertoire to a biased adult KIR repertoire and to elucidate how different KIR-HLA combinations contribute to the control of neonatal infections. Immunotherapy using NK-enhancing cytokines like IL-15 may benefit neonates with severe viral infections.
